# The Pathophysiology of Post-Traumatic Glioma

**DOI:** 10.3390/ijms19082445

**Published:** 2018-08-19

**Authors:** Donata Simińska, Klaudyna Kojder, Dariusz Jeżewski, Ireneusz Kojder, Marta Skórka, Izabela Gutowska, Dariusz Chlubek, Irena Baranowska-Bosiacka

**Affiliations:** 1 Department of Biochemistry and Medical Chemistry, Pomeranian Medical University, Powstańców Wlkp. 72, 70-111 Szczecin, Poland; d.siminska391@gmail.com (D.S.); dchlubek@sci.pam.szczecin.pl (D.C.); 2 Department of Anesthesiology and Intensive Care, Pomeranian Medical University in Szczecin, 71-252 Szczecin, Poland; klaudynakojder@gmail.com; 3 Department of Neurosurgery and Pediatric Neurosurgery, Department of Applied Neurocognitivistics, Pomeranian Medical University in Szczecin, 71-252 Szczecin, Poland; djezewski@wp.pl (D.J.); i.kojder@gazeta.pl (I.K.); 4 Department of Biochemistry and Human Nutrition, Pomeranian Medical University in Szczecin, 71-252 Szczecin, Poland; marta_skorka@o2.pl (M.S.); izagut@poczta.onet.pl (I.G.)

**Keywords:** injury, pathophysiology, brain, blood–brain barrier, post-traumatic glioma, IL-6, STAT3

## Abstract

Malignant glioma is a brain tumor with a very high mortality rate resulting from the specific morphology of its infiltrative growth and poor early detection rates. The causes of one of its very specific types, i.e., post-traumatic glioma, have been discussed for many years, with some studies providing evidence for mechanisms where the reaction to an injury may in some cases lead to the onset of carcinogenesis in the brain. In this review of the available literature, we discuss the consequences of breaking the blood–brain barrier and consequences of the influx of immune-system cells to the site of injury. We also analyze the influence of inflammatory mediators on the expression of genes controlling the process of apoptosis and the effect of chemical mutagenic factors on glial cells in the brain. We present the results of experimental studies indicating a relationship between injury and glioma development. However, epidemiological studies on post-traumatic glioma, of which only a few confirm the conclusions of experimental research, indicate that any potential relationship between injury and glioma, if any, is indirect.

## 1. Introduction

**Figure 1:**
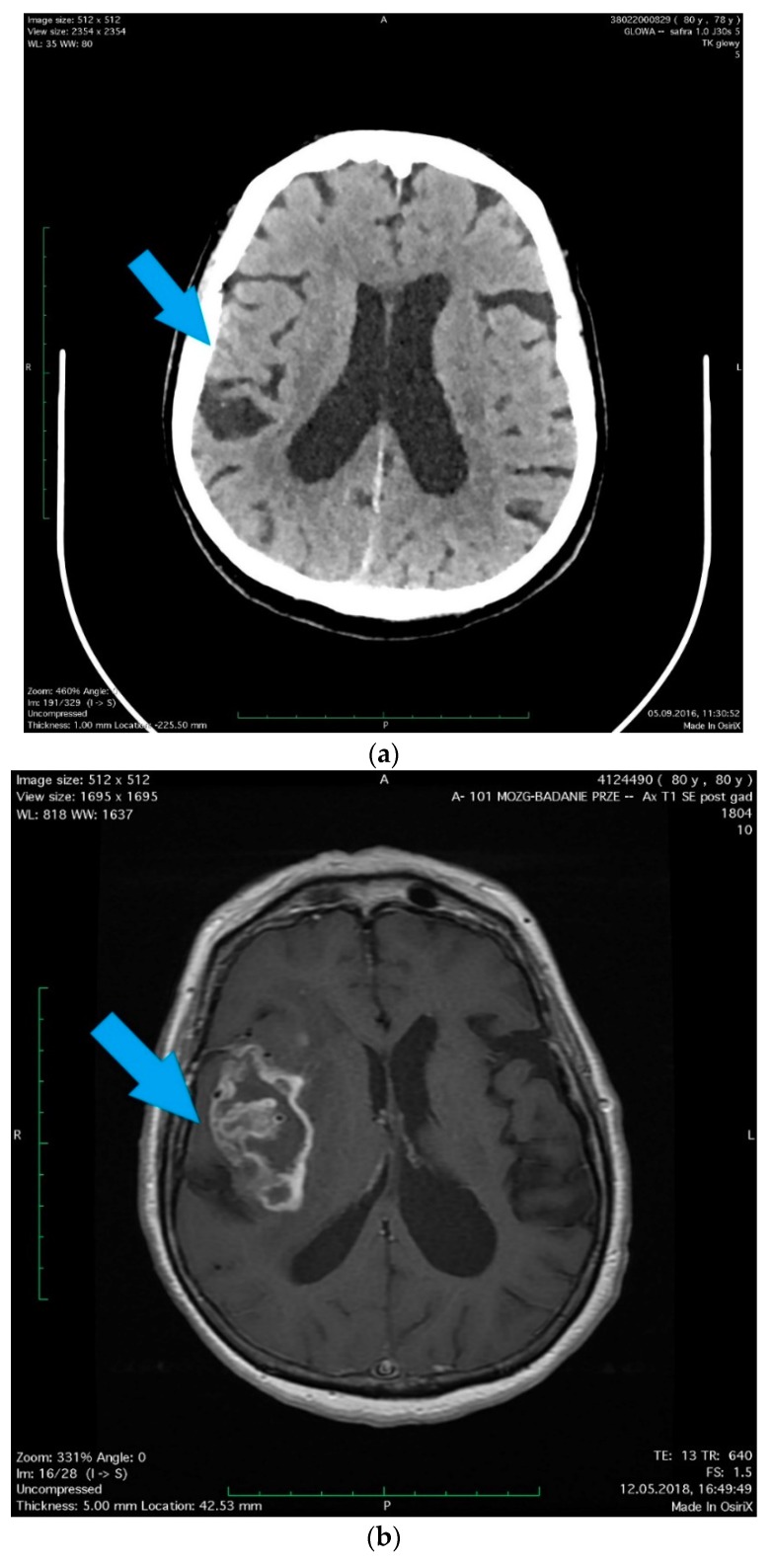

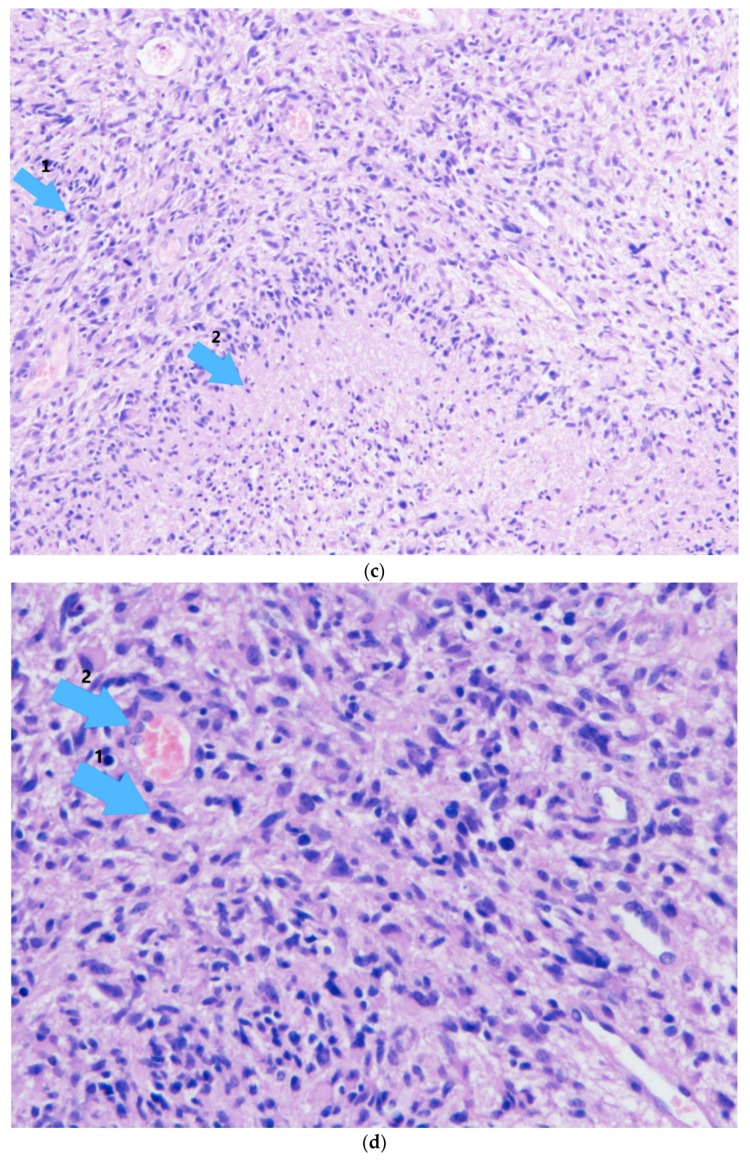
A 78-year-old woman with hypertension and hypercholesterolemia was admitted to the hospital due to her head injury (after falling in moving bus), small changes were revealed in the computed tomography (CT) scan as well as in the radiographic examination that did not require surgical treatment. (**a**) (arrow) A little amount of blood along the falx of the brain, on the tent of the cerebellum on the right side and in a fissura of the right parietal lobe with scant concussion in it. In the white matter of corona radiata, a number of dimly defined, diminished-density areas were observed, most likely corresponding to degenerative vascular changes. The patient, without neurological deficits except diminishing post-traumatic headaches, was discharged after the observation period in a good condition. Two years later, the patient was readmitted to the Department of Neurosurgery with a diagnosed brain tumor, manifested by a 1.5-month history of memory and orientation disorders. (**b**) (arrow) In the MRI scans, the right brain hemisphere tumor was found, the position of which corresponded to previous post-traumatic lesions. Right temporoparietal craniotomy was performed and the cytoreduction of the tumor was extended. Histopathology revealed Glioblastoma multiforme (facultatively). The histopathological pictures show (**c**) characteristic necrosis, surrounded by a pseudopalisade (arrow 1), visible cell mitosis (arrow 2), (**d**) large cell density, mitosis (arrow 1), lumen of the vessel with erythrocytes (arrow 2). The patient was accepted for radiotherapy with chemotherapy and discharged home in good condition, without additional neurological deficits, walking with little assistance. The study was approved by the Local Ethical Committee at the Pomeranian Medical University in Szczecin, Poland (approval No. KB-0012/96/14; Szczecin, Poland, 24.11.2014).

One of the most serious brain tumors is the central-nervous-system glioma. Characterized by high morphological and genetic complexity, it is formed as a result of neoplastic transformation of glial cells. At the time of diagnosis of glioblastoma multiforme (GBM), the average life expectancy, despite multidirectional therapy, is only 14 months [1]. Every year, about 10,000 new cases of GBM are detected in the United States alone [2]. Gliomas account for approximately 28% of all brain tumors and 80% of primary malignant brain tumors [3,4], with incidence estimated at 5.42 per 100,000 people in the 0–19 age group [3]. GBM constitutes about one-quarter of all gliomas, with incidence estimated at about 3/100,000 per year [3]. As statistical reports on brain tumors do not include post-traumatic glioma, it is difficult to find reliable information on the incidence of gliomas caused by traumas. Information on their prevalence can be derived from epidemiological studies of post-traumatic gliomas. In the study by Munch et al. [5], the incidence of post-traumatic glioma among patients who had suffered from some kind of brain trauma was established at 3/10,000. However, this result was determined only for a particular test group and should not be translated into the entire population. In addition, the incidence of post-traumatic glioma is difficult to establish due to the frequently considerable time gap between injury and glioma. Cancer cells of the glioblastoma multiforme spread by infiltrating into the adjacent tissues [1]. This is a serious limitation of any surgical treatment and often results in the removal of some healthy tissue during surgery. Yet, despite the use of techniques such as fluorescent labeling, there is still a high risk of leaving some of the tumor in the brain. This is a significant problem during tumor resection, one of the main current methods of glioma treatment, apart from chemotherapy and radiotherapy. Although new treatment techniques, such as immunotherapy or gene therapy, are being developed and are slowly introduced, treating glioblastoma is still a major challenge to modern medicine [1]. There are few documented cases in which the relationship between brain injury and subsequent glioma can be demonstrated. Uncertainty regarding this issue has not been resolved for a century [6]. However, tests and case studies needed some criteria for a GBM tumor to be considered post-traumatic glioma. The first guidelines in this field were proposed as early as the pre-computed tomography (CT) era by Zulch in 1965, and then by Manuelidis in 1972—both described in the study by Zhou [7]. Those researchers identified the following criteria for post-traumatic glioma: (1) The patient should be in good health before being injured; (2) damage must be serious enough to cause brain injury and a secondary repair process; (3) tumor and injury location must be the same; (4) both the tumor and traumatic brain environment should be confirmed histopathologically; (5) the interval between injury and the onset of the tumor should be more than a year—the later the onset, the greater the cause–effect relationship; (6) the trauma should arise from an external force; (7) bleeding, scars, and secondary edema must be characterized as being induced by the tumor, not the injury; (8) tumor tissue should be a direct continuity of the traumatic scar, and not only be in its vicinity or separated by a healthy zone [7]. After 1972, routine use of CT and then nuclear magnetic resonance (NMR) began for medical examination of patients, which opened new possibilities in the diagnosis of post-traumatic glioma. Table 1 presents a short description of a patients with post-traumatic glioma diagnosed with the aforementioned methods (Table 1 and Figure 1). In connection with the development of technology, researchers could come up with additional criteria for imaging brain areas immediately after the trauma, and the tumor formed at the same site. Criteria established for the identification of post-traumatic glioma became less stringent, and began to include the results of imaging tests [8,9]. However, there have been problems with finding fully documented cases due to the difficulty with meeting all guidelines, including the lack of histopathological confirmation of injury.

## 2. Brain Injury and Repair Processes

An important consideration for the recognition of malignant post-traumatic glioma is the pathophysiology and scope of documented mechanisms that may have contributed to its formation. Experimental studies, as well as observations of patients with brain injuries followed by glioma, have proposed several mechanisms that are highly likely associated with the formation of this trauma-induced malignant tumor. As it has been shown to date, the formation of glioma after injury may be associated with the inflammatory processes of the injury, whose main purpose is the elimination of damaged tissue components and the resumption of physiological activities [2]. In the entire body, this task is performed by the cells of the immune system that exist in the tissues and circulate in the blood; in the brain this role is specifically played by the microglia, in some cases aided by other cells of the immune system [2,10]. Injury-induced activation of microglia and the immune system is accompanied by several processes caused by external tissue damage, such as a hemorrhage, cerebral edema, and increased intracranial pressure—all related to the disruption of the brain’s protective and stabilizing structures and the blood–brain barrier [10]. Brain-repair processes in physiological conditions involve only microglia, usually isolated from other cells that perform repair functions, such as neutrophils and macrophages. However, at the time of injury this isolation is compromised and the cells of the immune system enter the parenchyma of the brain along with blood. This process becomes permanent because the interruption of the blood–brain barrier is caused not only by mechanical injury of the vessel, but is also induced by hypoxia and subsequent acidosis [11].

## 3. The Role of IL-6 Secreted by Macrophages

Macrophages flowing to the site of the traumatically interrupted blood–brain barrier are activated to monocytes and secrete interleukin 6 (IL-6), a compound which is also secreted by T and B lymphocytes, endothelial cells, and in the brain mainly produced by astrocytes and glial cells. IL-6 is a 26 kDa protein that consists of 212 amino acids. This cytokine with an immunomodulatory function is present in the brain also under physiological conditions, regulating various physiological processes in the immune response of the central nervous system (CNS). However, in normal conditions its expression is very low. Injury induces an increased IL-6 production by glial cells in the CNS. In addition, it is also secreted by the peripheral cells of the immune system flowing in with the blood, which causes a significant increase in its concentration at the site of injury. In addition, IL-6 in larger quantities adversely affects the microcirculation in the brain, increasing vascular permeability, and damaging the blood–brain barrier in a much wider area than the initial area. This function is very important in injuries that did not initially break the barrier because IL-6 allows the secondary entry of macrophages to the site of injury, and also exacerbates its course by creating brain edema [2,10]. IL-6 also plays a function in cell-cycle regulation processes, as has been proven in experimental studies in rats [10]. It activates signal transducer and activator of transcription 3 (STAT3), a protein responsible for cell growth, proliferation, differentiation, and apoptosis. Studies have shown that inhibition of STAT3 suppresses the growth and proliferation of glioblastoma cells and also induces their apoptosis [1]. This has been confirmed in both in vitro and in vivo studies [12,13]. IL-6 secreted by macrophages activates this protein, thus inhibiting apoptotic processes and increasing the proliferation of cells located at the site of injury. In addition, the active STAT3 has been shown to inhibit the cellular response of T lymphocytes, and also to reduce the activity of major histocompatibility complex (MHC) class II molecules on microglial cells and other antigen–presenting cells of hematopoietic origin [1]. It is possible, then, that the STAT3 protein has an immunosuppressive effect. This is a reason to consider it a carcinogenic factor for glioma, which, when combined with an injury, may support the view of induction of this tumor. Another argument supporting the thesis of the pathophysiological role of IL-6 in the mechanism of glioma formation is its high concentrations and its receptors in cerebral spinal fluid in patients after head injury. Its high levels may cause the production of proinflammatory cytokines that induce an elevated inflammatory response in the body [10]. Although IL-6 is important for the proper functioning of the brain regardless of injury, and the inflammatory response itself is necessary for the proper healing process, excessive IL-6 concentrations can cause negative effects. Therefore, it seems reasonable to try to reduce its amount after injury to protect cells from its adverse effects. Such attempts are already being made using modern gene-therapy techniques [10].

## 4. The Role of Eosinophil Peroxidase and Reactive Oxygen Species (ROS)

Eosinophils, which flow through the damaged barrier, secrete eosinophil peroxidase, which generates ROS [2]. Although ROS induce apoptosis of cells, their amount is apparently not sufficient to apoptose intensely proliferating microglia and macrophages, forming about 1/3 of the glial tumor mass [1,14,15]. Instead, due to the well-known ability of ROS to induce mutations that fundamentally change the functions of microglia, intensive proliferation of eosinophils induced by substances secreted by other cells of the immune system may contribute to the development of a cancer-transformed cell line. 

## 5. Inflow of Stem Cells

The subependymal zones of the lateral ventricles and the subgranular zone of the hippocampal dentate gyrus of the adult brain contain stem neuronal cells, similar to the olfactory epithelium [2]. Brain injury, in addition to the influx of immune cells, causes the migration of neuronal stem cells and progenitor cells to the site of injury. These cells migrate topically to damaged areas and start the regeneration process, differentiating into neurons, astrocytes, and oligodendrocytes. However, they also show increased expression of oncogenic genes, decreased suppressor-gene activity, and high sensitivity to chemical mutagenic factors [2]. In addition, progenitor cells initiating a brain tumor in situations induced by stroke- or trauma-related ischemia may result in the formation of astrocytes with altered activity [16,17]. The resultant excessive production of glutamate or STAT3 by astrocytes in the glioma suppresses the immune response [18,19]. Sensitivity to mutagenic factors revealed by stem cells is very important just at the time of injury and the moment of penetration of immune cells into the brain, because then stem cells are accompanied by proinflammatory factors and ROS produced by eosinophils, as explained earlier [2]. Neuronal stem cells are highly sensitive to mutagenic agents and are easily mutated as a result of their action. Moreover, the reduced activity of suppressor genes limits the ability of these cells to defend against neoplastic transformation. These characteristics of neuronal stem cells may increase the mutation frequency and the formation of rapidly proliferating tumor cells, which increase the tumor mass not only by division, but also by inducing the formation of a tumor microenvironment that further promotes the development of cancer [2,20]. The migration of stem cells has been confirmed in cases of ischemia, demyelination, and trauma. However, only in trauma are these cells exposed to the aforementioned factors and only in trauma is there such a high risk of induction of neoplastic transformation [2]. Therefore, it seems reasonable to associate an injury that induces brain stem cell activity with a subsequent development of neoplasm [2]. Stem cells, not only from the subependymal area but also from other locations, are recognized as potentially oncogenic in GBM [8]. Some researchers argue that they play this role in all gliomas [21,22,23]. Our own observations in a female patient with recurrent glioma in the occipital lobe revealed changes in the density of the white matter of the brain, spreading radially from the tumor bed in the period preceding the formation of GBM [24]. As surgical procedure included all elements of cerebral trauma, it could be considered as stimulating tumor growth. Corrective action of stem cells usually concerns tissues adjacent to the tumor bed. The surgical trauma, although limited initially to the precise interruption of the brain–blood barrier, has all the other traits of an unintended injury. Thus, it is an iatrogenic model of injury with all its consequences, and causes a decrease in the immunological suppression of tumor growth [25]. Craniocerebral injuries have long been considered a cause associated with the development of cancer. At the beginning, mainly meningiomas were associated with the post-traumatic translocation of cells outside the natural site. Later, gliomas were also associated with trauma. The description of the role of stem cells and consequences in the light of these observations should be taken into account in the analysis of a pathological process leading to the onset of a post-traumatic GBM. Stem cells used for restoration of post-stroke areas may have tumorigenic potential and other undesirable effects including molecular effects that may trigger further adverse effects and affect higher neurological activities.

Restoration involving stem cells usually occurs in the tissue surrounding the tumor bed. The surgical trauma, although initially limited to the brain–blood barrier, has all the other traits of an unintended injury. Thus, it is an iatrogenic model of injury with all its consequences, resulting in decreased immunosuppression of the tumor.

Although there is no literature on this subject, there are some reports on the development of glioblastoma multiforme caused by irradiation following surgical trauma associated with biopsy or cytoreduction [25], even after the intraoperatively and radiologically verified complete removal of the tumor [26].

## 6. The Role of Microglia Cells

Microglia in the brain perform a function similar to the function of peripheral macrophages. Their roles include phagocytosis, antigen presentation, and the release of cytokines and chemokines [1]. In most cases this is how microglia function, securing the area separated from the peripheral immune system by the blood–brain barrier. Cytotoxic effects in this respect have been confirmed in vitro [1]. However, in vivo studies show that microglia have a different effect on the development of brain glioma: they produce immunosuppressive conditions that allow cancers to grow [27,28]. While the inductive role of microglia in the growth of post-traumatic glioma is debatable, its role in creating an environment facilitating development has been confirmed [1]. In the tissues adjacent to glioma, microglia do not release proinflammatory cytokines, but produce metalloproteinases that facilitate tumor invasion [27]. PGE2 prostaglandins also participate in the creation of conditions conducive to the development of glioma. They are synthesized by microglia accompanying the developing glioblastoma and suppress the activation of T lymphocytes and their proliferation, as well as decrease the expression of MHC class II molecules on antigen-presenting cells. PGE2 creates an immunosuppressive environment that facilitates further development of glioma [29].

## 7. Interruption of the Blood–Brain Barrier

Interruption of the blood–brain barrier is not always the result of a mechanical injury. It also occurs during infection due to the action of proinflammatory prostaglandins, thromboxanes, and leukotrienes, affecting the blood–brain barrier and triggering the effect of relaxation of tight junctions (TJ) [11,30]. Under the influence of these proinflammatory factors, the capillary epithelium also relaxes and thus glial cells become exposed to potentially mutagenic substances that would not reach them under physiological conditions [30]. Brain injury accompanied by a cascade of blood–brain-barrier damage and neurobiochemical consequences of blood extravasation always cause a recovery reaction. This explains how cancer recurrence in some patients. In light of the above, a question that remains is why the majority of patients do not develop cancer after trauma, and what the role of surgery in the process of cancer recurrence is.

**Figure 2:**
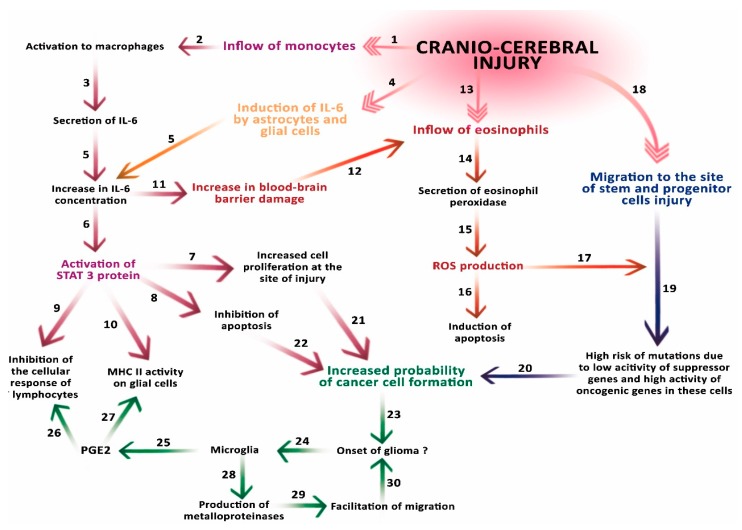
Schematic representation of the involvement of carcinogenic factors after cerebral injury. An injury causes the influx of monocytes to the site of damage (1), where they are activated to macrophages (2) that produce interleukin 6 (IL-6) (3). The injury also induces enhanced IL-6 secretion by astrocytes and microglial cells (4). Together, these processes cause a significant increase in IL-6 concentration (5). The increased concentration of IL-6 activates STAT3 (6), which increases cell proliferation at the site of injury (7) and inhibition of apoptosis (8). STAT3 inhibits the activity of T cells (LcT) and (9) decreases the activity of major histocompatibility complex (MHC) particles on cells of the immune system and glial cells (10). The increase in IL-6 concentration also affects the blood–brain barrier (BBB) (11), facilitating the penetration by eosinophils (12) flowing to the site of injury (13). The eosinophils, which flow through the damaged barrier, secrete eosinophil peroxidase, which generates reactive oxygen species (ROS). ROS contribute to the induction of apoptosis (16), but may also contribute to the formation of mutations (17) in stem and progenitor cells that migrate to the site of injury (18). Low activity of suppressor genes in these cells and high activity of oncogenic genes may additionally increase the risk of mutations (19). The risk of these mutations (20), an increase in cell proliferation at the site of injury (21) and the inhibition of apoptosis (22) may jointly contribute to the formation of a cancer cell, and start the process of carcinogenesis (23). The glioma resulting from these processes affects microglia (24), which secrete PGE2 (25), increasing the inhibition of LcT activity (26) and causing a decrease in MHC molecules activity on cells of the immune system and glial cells (27). In addition, microglia secrete metalloproteinases (28) in the tissues adjacent to the tumor, facilitating its migration (29) and thus facilitating its development (30).

All the mechanisms presented in Figure 2 are described in this article.

## 8. Epidemiological and Experimental Research on Post-Traumatic Glioma

Although the reports from epidemiological observations, including cohort studies, mentioned in the literature do not explicitly confirm the relationship between brain trauma and glioblastoma [2,31,32,33], there are reports suggesting the possibility of such a relationship [2,9,34,35,36,37].

The results of the presented epidemiological studies cannot be compared uncritically, because they concern different ethnic groups, people of different ages, living in different environments. They have also not been standardized in any way as to the type and severity of brain damage. Difficulties in this type of research result, among other things, from the low incidence of brain tumors. There is still a need for large-scale and comprehensive epidemiological investigations for post-traumatic glioma, which is supported by a fairly large number of reports [2,7,9,33,38,39,40].

## 9. Strategy for Further Study and at the Clinical Approach

Although the widespread occurrence of craniocerebral traumas without subsequent GBM suggests the influence of an unknown agent, it is also possible that all GBM patients have had some kind of brain trauma in the past (not only craniocerebral), which should then be the subject of retrospective and prospective verification, complemented by a detailed whole-brain autopsy. Another important issue is the role of brain surgery, which could be established by comparing growth dynamics between post-traumatic tumors following a surgery varying in scope and location, and tumors in those GBM patients who have not been operated on. Another potential area of interest is the potentially carcinogenic migration of different tissues, e.g., whole-blood or morphotic-blood elements, into neural-tissue parenchyma. Finally, the post-traumatic shift of meningioma to areas distant from their origin has also been implicated in carcinogenesis. 

All the aforementioned questions show the necessity of comprehensive research on the relation between post-traumatic mechanisms and tumorigenesis. However, this research is burdened with considerable obstacles. The considerable time interval between trauma and tumor onset makes it difficult to perform adequate in vivo tests on animals. In vitro tests on primary cultures are also problematic due to the large number of different types of cells constituting the brain tissue and their metabolic cooperation. The use of immortalized glial cell lines is also excluded due to their physiological dissimilarity to normal brain cells. Therefore, we are still in need of effective in vivo and in vitro models that could help in explaining the mechanisms behind the formation of the post-traumatic GBM.

## 10. Conclusions

In addition to epidemiological research, there are also experimental studies to investigate the pathophysiology of glioma and confirm correlations between injury and glioma in laboratory conditions. These studies, in contrast to epidemiological studies, do provide evidence of a relationship between brain injury and glioma. The reasons for this discrepancy were analyzed by Zhou et al. who concluded that the lack of connection between injury and glioma in epidemiological studies indicate additional factors involved in the formation of the post-traumatic glioma [7]. These factors may be unintentionally omitted during the selection of study groups, which is why further studies on the etiology of post-traumatic glioma are so important.

## Figures and Tables

**Table 1:** A case of a post-traumatic glioma.

**Sex**	Woman
**Age**	78-year-old
**Comorbidity**	hypertension and hypercholesterolemia
**Reason for admission to the hospital**	head injury (after falling in moving bus)
**CT test result**	A little amount of blood along the falx of the brain, on the tent of the cerebellum on the right side in a fissure of the right parietal lobe with scant concussion in it.
	The patient, without neurological deficits, except diminishing post-traumatic headaches, was discharged after the observation period in a good condition
**Readmission to the hospital**	2 years later
**Reason for readmission to the hospital**	diagnosed brain tumor
**Symptoms**	1.5-month history of memory and orientation disorders
**MRI test result**	the right brain hemisphere tumor was found, the position of which corresponded to previous post-traumatic lesions
**Treatment**	right temporoparietal craniotomy was performed and the cytoreduction of the tumor was extended
**Changes in the histopathological picture**	characteristic necrosis, surrounded by a pseudopalisade, visible cell mitosis, large cell density, mitosis, lumen of the vessel with erythrocytes
**Histopathological result**	Glioblastoma multiforme
**Further treatment**	radiotherapy with chemotherapy
